# Supplementary material for: Causal Relationship between Aging and Anorexia Nervosa: A White-Matter-Microstructure-Mediated Mendelian Randomization Analysis
Source: Biomedicines. 2024 Aug 16;12(8):1874. doi: 10.3390/biomedicines12081874 (PMC11351342; doi:10.3390/biomedicines12081874)
Supplement: Supplementary file 1 [file biomedicines-12-01874-s001.zip › Supplementary Figure/Figure S4/Singe SNP plot of MR analysis for TL to ubm-b-1535.pdf]

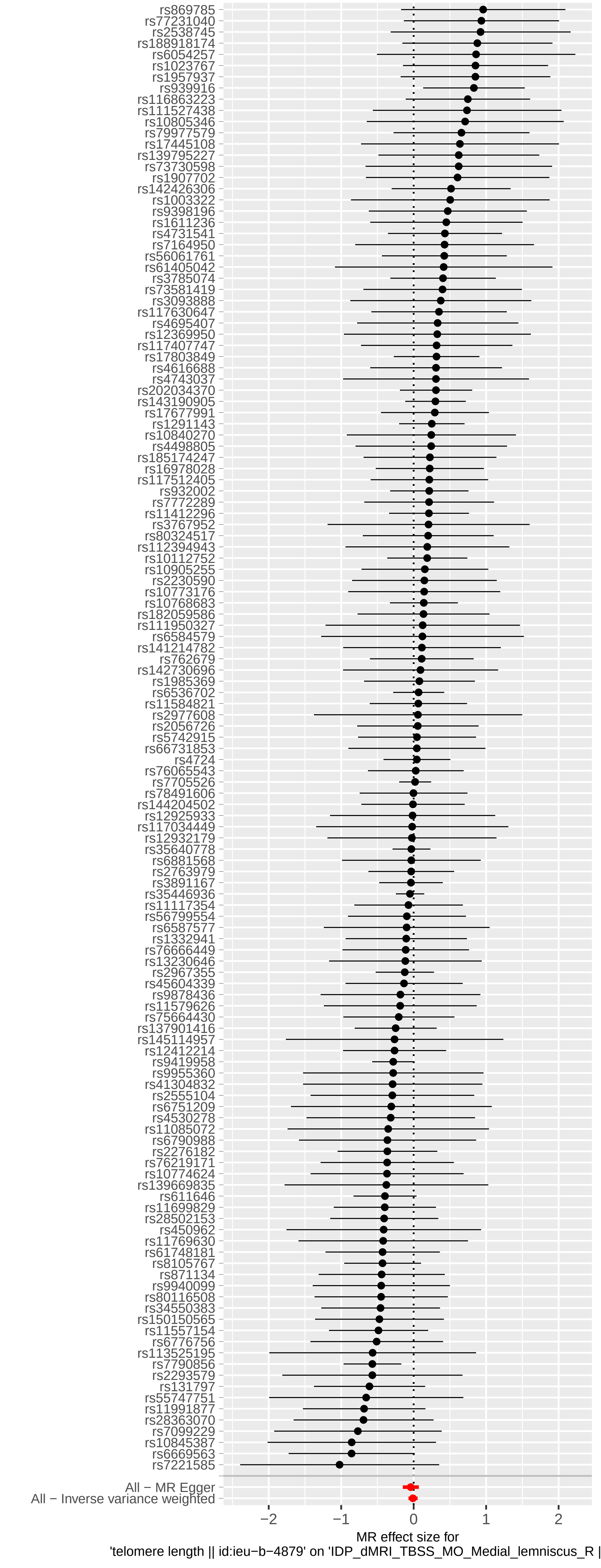
rs869785
rs77231040
rs2538745
rs188918174
rs6054257
rs1023767
rs1957937
rs939916
rs116863223
rs111527438
rs10805346
rs79977579
rs17445108
rs139795227
rs73730598
rs1907702
rs142426306
rs1003322
rs9398196
rs1611236
rs4731541
rs7164950
rs56061761
rs61405042
rs3785074
rs73581419
rs3093888
rs117630647
rs4695407
rs12369950
rs117407747
rs17803849
rs4616688
rs4743037
rs202034370
rs143190905
rs17677991
rs1291143
rs10840270
rs4498805
rs185174247
rs16978028
rs117512405
rs932002
rs7772289
rs11412296
rs3767952
rs80324517
rs112394943
rs10112752
rs10905255
rs2230590
rs10773176
rs10768683
rs182059586
rs111950327
rs6584579
rs141214782
rs762679
rs142730696
rs1985369
rs6536702
rs11584821
rs2977608
rs2056726
rs5742915
rs66731853
rs4724
rs76065543
rs7705526
rs78491606
rs144204502
rs12925933
rs117034449
rs12932179
rs35640778
rs6881568
rs2763979
rs3891167
rs35446936
rs11117354
rs56799554
rs6587577
rs1332941
rs76666449
rs13230646
rs2967355
rs45604339
rs9878436
rs11579626
rs75664430
rs137901416
rs145114957
rs12412214
rs9419958
rs9955360
rs41304832
rs2555104
rs6751209
rs4530278
rs11085072
rs6790988
rs2276182
rs76219171
rs10774624
rs139669835
rs611646
rs11699829
rs28502153
rs450962
rs11769630
rs61748181
rs8105767
rs871134
rs9940099
rs80116508
rs34550383
rs150150565
rs11557154
rs6776756
rs113525195
rs7790856
rs2293579
rs131797
rs55747751
rs11991877
rs28363070
rs7099229
rs10845387
rs6669563
rs7221585
All − MR Egger
All − Inverse variance weighted
−2
−1
0
1
2
MR effect size for
'telomere length || id:ieu−b−4879' on 'IDP_dMRI_TBSS_MO_Medial_lemniscus_R |
